# Lipid Dynamics in Diisobutylene-Maleic Acid (DIBMA) Lipid Particles in Presence of Sensory Rhodopsin II

**DOI:** 10.3390/ijms22052548

**Published:** 2021-03-04

**Authors:** Natalia Voskoboynikova, Philipp Orekhov, Marine Bozdaganyan, Felix Kodde, Malte Rademacher, Maurice Schowe, Annette Budke-Gieseking, Britta Brickwedde, Olympia-Ekaterini Psathaki, Armen Y. Mulkidjanian, Katia Cosentino, Konstantin V. Shaitan, Heinz-Jürgen Steinhoff

**Affiliations:** 1 Department of Physics, University of Osnabrück, Barbarastrasse 7, D-49076 Osnabrück, Germany; nvoskobo@uni-osnabrueck.de (N.V.); fkodde@outlook.de (F.K.); mrademacher@uni-osnabrueck.de (M.R.); mschowe@uni-osnabrueck.de (M.S.); amulkid@uni-osnabrueck.de (A.Y.M.); 2 Moscow Institute of Physics and Technology, 141701 Dolgoprudny, Russia; ps.orekhov@gmail.com; 3 Institute of Personalized Medicine, Sechenov University, 119146 Moscow, Russia; 4 Department of Biology, Lomonosov Moscow State University, 119991 Moscow, Russia; m.bozdaganyan@gmail.com (M.B.); shaytan49@yandex.ru (K.V.S.); 5 N.N. Semenov Federal Research Center for Chemical Physics, Russian Academy of Sciences, 119991 Moscow, Russia; 6 Department of Biology and Center for Cellular Nanoanalytics (CellNanOs), University of Osnabrück, Barbarastrasse 11, D-49076 Osnabrück, Germany; annbudke@uni-osnabrueck.de (A.B.-G.); bbrickwedde@uni-osnabrueck.de (B.B.); katherina.psathaki@uni-osnabrueck.de (O.-E.P.); kacosentino@uni-osnabrueck.de (K.C.); 7 Faculty of Bioengineering and Bioinformatics and Belozersky Institute of Physico-Chemical Biology, M.V. Lomonosov Moscow State University, 119234 Moscow, Russia

**Keywords:** nitroxide spin label, electron paramagnetic resonance spectroscopy (EPR; ESR), polymer/lipid nanodiscs, phospholipid bilayer, membrane protein, negative-stain and cryo-transmission electron microscopy (EM), coarse-grained (CG), molecular dynamics (MD)

## Abstract

Amphiphilic diisobutylene/maleic acid (DIBMA) copolymers extract lipid-encased membrane proteins from lipid bilayers in a detergent-free manner, yielding nanosized, discoidal DIBMA lipid particles (DIBMALPs). Depending on the DIBMA/lipid ratio, the size of DIBMALPs can be broadly varied which makes them suitable for the incorporation of proteins of different sizes. Here, we examine the influence of the DIBMALP sizes and the presence of protein on the dynamics of encased lipids. As shown by a set of biophysical methods, the stability of DIBMALPs remains unaffected at different DIBMA/lipid ratios. Coarse-grained molecular dynamics simulations confirm the formation of viable DIBMALPs with an overall size of up to 35 nm. Electron paramagnetic resonance spectroscopy of nitroxides located at the 5th, 12th or 16th carbon atom positions in phosphatidylcholine-based spin labels reveals that the dynamics of enclosed lipids are not altered by the DIBMALP size. The presence of the membrane protein sensory rhodopsin II from *Natronomonas pharaonis* (*Np*SRII) results in a slight increase in the lipid dynamics compared to empty DIBMALPs. The light-induced photocycle shows full functionality of DIBMALPs-embedded *Np*SRII and a significant effect of the protein-to-lipid ratio during preparation on the *Np*SRII dynamics. This study indicates a possible expansion of the applicability of the DIBMALP technology on studies of membrane protein–protein interaction and oligomerization in a constraining environment.

## 1. Introduction

Interactions of integral membrane proteins trigger a variety of processes in cellular membranes making important contributions to cell function and having a high pharmacological importance [1]. Application of various biophysical methods to the study of membrane protein structure and dynamics generally requires the preparation of water-soluble membrane protein samples in a native-like environment. Membrane proteins are usually studied in vitro by incorporating them into micelles, liposomes or nanolipoprotein particles (NLPs or nanodiscs) [2,3] with the help of detergents, which can negatively affect the stability and activity of proteins [4,5,6]. An alternative approach is to use amphiphilic styrene maleic acid (SMA) copolymers, which then form soluble SMA-lipid particles (SMALPs). SMA copolymers have been shown to solubilize membrane proteins by direct extraction from natural lipid bilayers or artificial membranes without applying detergents during any of the preparation steps ([7,8,9,10], reviewed, e.g., in [11,12,13,14,15]). The SMA copolymer-driven lipid solubilization also has the advantage of being non-selective concerning the lipid type [16,17]. The size of these nanoparticles is about 10 nm depending on the preparation routine [18]. The small size and single-particle character make SMALPs suitable for investigation of membrane proteins and their interactions [19] by spectroscopic techniques and also by electron microscopy (EM) and, in particular, by cryo-EM [20,21,22,23].

Recently, Keller and colleagues have shown that the diisobutylene/maleic acid (DIBMA) copolymers, which contain aliphatic diisobutylene styrene groups instead of aromatic ones, can, similar to SMA, solubilize lipids without using detergents resulting in the formation of stable DIBMA/lipid particles (DIBMALPs) somewhat larger than SMALPs [24,25]. Furthermore, the size of DIBMALPs can be varied in a broader range than that of SMALPs and monotonously decreases with an increasing polymer/lipid ratio [24,25]. Therefore, the DIBMA-based approach could be advantageous for the study of large membrane proteins or membrane protein complexes, as well as their interactions, by providing a higher degree of freedom for protein conformational changes or protein–protein interaction. Membrane proteins enclosed in stable polymer-stabilized lipid particles can be studied by different biophysical methods without loss of their structural and functional properties [13,26,27].

Membrane proteins and their function could be influenced by their lipid surroundings [28,29,30]. Therefore, investigations of membrane proteins in a new lipid model system such as DIBMALPs require understanding of the properties of the DIBMA-enclosed lipid bilayer to confirm its biological relevance. However, it has remained unclear whether the resizing of DIBMALPs and the introduction of proteins can affect the enclosed lipid patch, thereby possibly influencing the structure, conformational stability, or dynamics of inserted proteins.

Dynamic properties of membrane proteins and lipid bilayers incorporated in liposomes, nanodiscs and SMALPs have been studied by electronic paramagnetic resonance (EPR) spectroscopy [9,19,31,32,33,34,35,36,37,38,39]. Upon introduction of spin probes into the proteins or lipids valuable structural and dynamic information were obtained [40,41,42]. Nitroxides attached to carbon atoms at different positions along the lipid acyl chain allow in-depth probing of the lipid bilayer in order to characterize the segmental chain mobility [42]; EPR combined with site-directed spin labeling reveals possible effects of confinement structure and dynamics of the encased proteins [9,38]. In addition, molecular dynamics (MD) simulations, including coarse-grained (CG) modeling, enable precise control over the content and simulated conditions of investigated systems and provide insights into the molecular mechanisms of lipid membrane solubilization and lipid nanoparticle stabilization by amphiphilic polymers [43]. For example, a recent CG MD simulation study examined the behavior of SMA copolymers with varying composition, charge and concentration in solution, as well as their interaction with lipid membranes [44].

In our previous work, we compared the dynamics of lipids in lipid nanoparticles formed by the SMA (3:1) and DIBMA copolymers using EPR spectroscopy and a set of phosphatidylcholine lipids with nitroxide groups introduced at different carbon atom positions [45]. Our results revealed that lipids are dynamically more constrained in SMALPs. Complimentary CG MD simulations indicated that, in the case of DIBMALPs, a single belt of polymer encircles lipids within the lipid nanoparticles and provided a possible explanation for the fact that lipid dynamics in DIBMALPs more closely resemble those in liposomes [45].

In the present work we focus on lipid and protein dynamics in DIBMALPs of different size prepared at different protein-to-lipid ratios. We apply EPR spectroscopy to study possible effects of varying sizes of DIBMALPs on the dynamics of enclosed lipids in the absence and presence of the membrane protein sensory rhodopsin II from *Natronomonas pharaonis* (*Np*SRII). To this end, we use a set of phosphatidylcholine lipids with nitroxide groups located at the 5th, 12th or 16th carbon atom positions along the hydrocarbon chain of the lipid and the saturated neutral lipid 2-dimyristoyl-*sn*-glycero-3-phosphocholine (DMPC). The EPR results show that neither the size of the nanoparticle nor the presence of the protein significantly alters the lipid dynamics in DIBMALPs. The light-induced photocycle of DIBMALP-embedded *Np*SRII reveals its full functionality, and the photocycle dependence on the protein-to-lipid ratio. Dynamic light scattering, electron microscopy and atomic force microscopy characterize the DIBMALP samples as homogeneous preparations of nanosized discoidal particles. Furthermore, CG MD simulations complement our experiments and show formation of stable DIBMALPs with an overall size up to 35 nm and uniform dynamics of the enclosed lipids.

## 2. Results

### 2.1. Biophysical Characterization of Empty and Protein-Containing DIBMALPs

**Figure 1:**
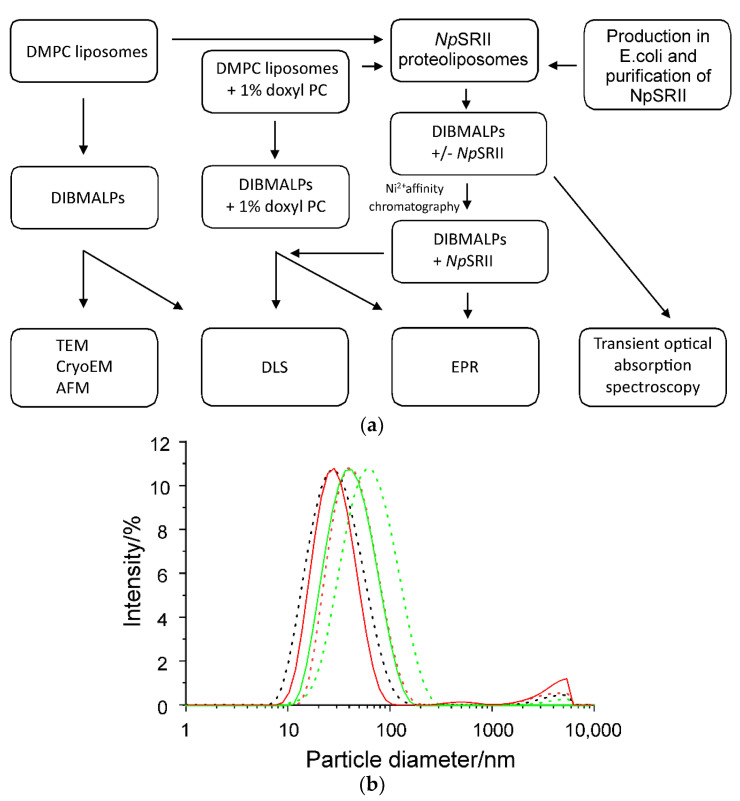
Characterization of lipid bilayer nanoparticles formed by diisobutylene/maleic acid (DIBMA). (**a**) Scheme showing the preparation of empty and protein-filled DIBMA lipid particles (DIBMALPs) and their biophysical characterization. (**b**) Representative dynamic light scattering (DLS) intensity-weighted size distributions of empty (broken lines) and *Np*SRII containing (continuous lines) DIBMALPs formed upon addition of 4% (*m*/*v*) DIBMA at a DIBMA/2-dimyristoyl-*sn*-glycero-3-phosphocholine (DMPC) weight ratio of 1:2 (green traces), 1:1 (red traces) and 2:1 (black traces) to DMPC liposomes. All intensities were normalized to that of the black trace.

The general scheme for the preparation of DIBMALP samples for further characterization is shown in Figure 1a. In a first step to characterize DIBMALPs of different sizes, we prepared DMPC liposomes and DMPC proteoliposomes containing *Np*SRII, and solubilized them with DIBMA copolymer at different DIBMA/DMPC weight ratios of 1:2, 1:1 or 2:1 (1:1 and 2:1 for the proteoliposomes) while keeping the lipid concentration constant (Figure 1a). At all selected DIBMA/lipid ratios, we could successfully obtain lipid nanoparticles.Dynamic light scattering (DLS) was employed to analyze the size distribution of particles in the preparations obtained upon lipid solubilization by the DIBMA copolymer. Figure 1b shows the DLS data for DIBMA/DMPC particles with or without protein after mixing of DMPC liposomes with 4% (*m*/*v*) DIBMA at different DIBMA/DMPC weight ratios. All samples show the unimodal character of intensity-weighted size distributions indicating the presence of monodisperse nanosized particles (Figure 1b). The significant shift of the respective size distributions of empty and protein filled DIBMALPs shows that not only the DIBMA/DMPC weight ratio but also the presence of the protein affects the average size of the DIBMALPs.

Table 1 summarizes the DLS data and shows that the overall size of DIBMALPs gradually decreases with increasing DIBMA/DMPC ratio. The measured z-average size of DIBMALPs at a DIBMA/DMPC weight ratio of 1:2 is approximately 54 nm. The size of particles decreases to around 42 nm at a DIBMA/DMPC weight ratio of 1:1 and decreases further to less than 30 nm at a DIBMA/DMPC weight ratio of 2:1. These results are consistent with previously published data [24,25]. The protein containing DIBMALPs are ~30% smaller.

**Figure 2:**
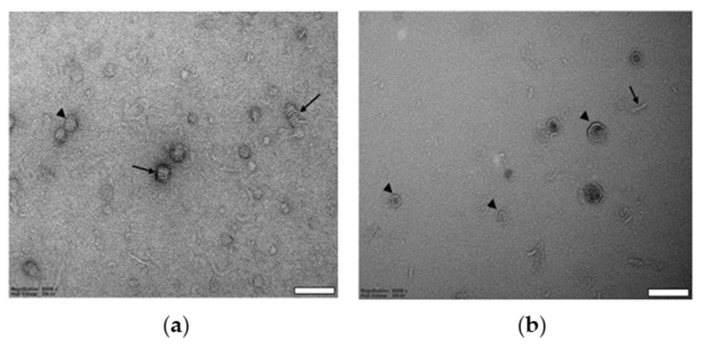
EM micrographs of DIBMA/DMPC nanoparticles. Negative-stain TEM (**a**) and cryo-TEM image (**b**) at 1:1 DIBMA/lipid weight ratio. The arrowheads and arrows indicate particles observed face-on and edge-on, respectively. Scale bar 100 nm.

To further validate individual DIBMALPs shapes, we performed EM visualization of negatively stained particle samples prepared at 1:1 polymer-to-lipid weight ratio and deposited on glow-discharged 400-mesh copper grids. TEM-micrographs show the individual DIBMALPs as disc-shaped single particles (indicated by arrowheads) as well as stacked flat discoidal structures (indicated by arrows) (Figure 2a). The sizes of both flat discs and stacked discs at negative-stained EM micrographs correspond to the DLS data. Further visualization of assembled DIBMALPs by cryo-TEM provided images of single particles, both face-on and edge-on, but not of such stacked discs as on EM-micrographs of negatively stained samples (Figure 2a,b). Thus, the EM data confirm the proper disc shapes of DIBMALPs in our preparations.

**Figure 3:**
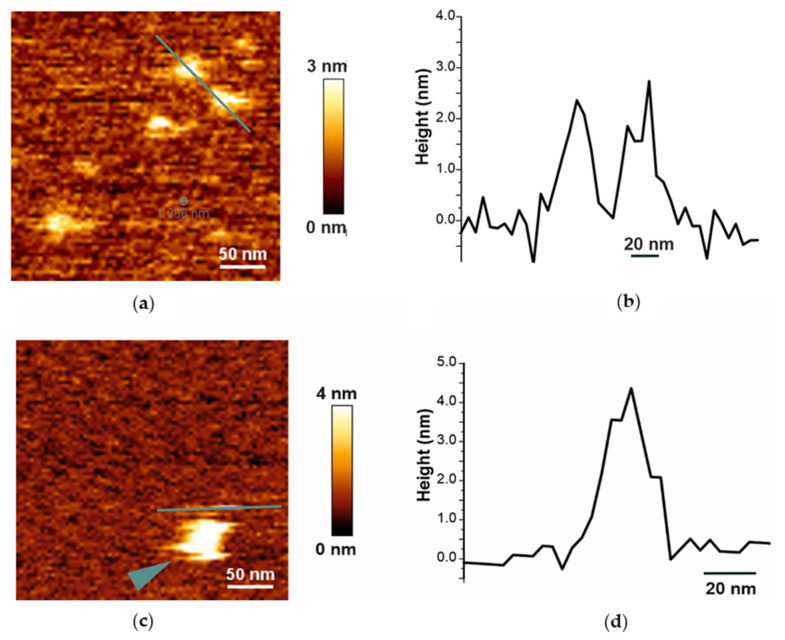
Atomic force microscopy (AFM) analysis of DIBMA/DMPC nanoparticles. (**a**,**c**) AFM images of DIBMA/DMPC nanoparticles prepared at 1:1 DIBMA/lipid weight ratio. The arrowhead indicates the presence of lipid nanoparticle stacks. (**b**,**d**) Height profile corresponding to the green line in the 2D image in (**a**,**c**) crossing individual lipid nanoparticles. The images are representative of at least two independent experiments.

Atomic force microscopy (AFM) was used for profiling the surfaces of assembled DIBMALPs. Like EM micrographs, solution AFM images of DIBMALP preparations at 1:1 polymer-to-lipid weight ratio show individual nanoparticles (Figure 3a) as well as stacked disc-shaped structures (Figure 3c). At the tested particles, the height of DIBMALPs (in respect to the mica support) corresponds to the expected lipid bilayer thickness of about 3.0–4.0 nm. These data are consistent with our previously published AFM results obtained for SMALPs [46] and confirm that the DIBMA belt supports the typical lipid-bilayer thickness.

### 2.2. Lipid Dynamics and Ordering Are Not Affected by the Presence of NpSRII

**Figure 4:**
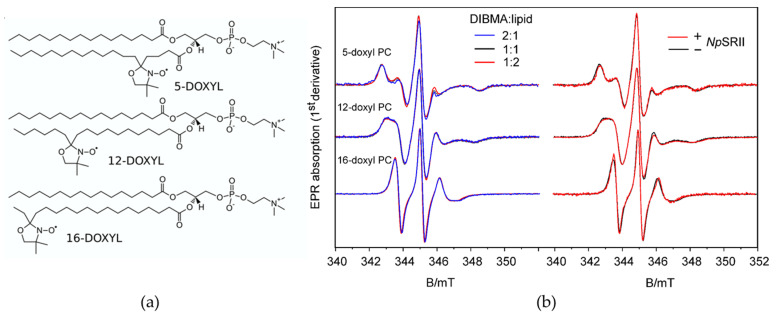
The dynamics of spin labeled lipids enclosed in DIBMALPs of different sizes and in absence and presence of *Np*SRII. (**a**) Doxyl-PCs with nitroxides bound at the 5th, 12th and 16th carbon position of the hydrocarbon chain. (**b**) EPR spectra recorded at room temperature for DIBMALPS prepared (left panel) with different DIBMA-to-lipid ratios (*w*/*w*), 2:1 (blue), 1:1 (black) and 1:2 (red), and (right panel) in presence (red) and absence (black) of *Np*SRII at a 1:1 (*w*/*w*) DIBMA-to-lipid ratio.

Using EPR spectroscopy we determined the dynamics of spin labeled lipids in DIBMALPs of different average sizes. The EPR line shape reflects the mobility of the nitroxide bound either to the 5th, 12th or 16th carbon atoms of the host phosphatidylcholine chain (in the following abbreviated as 5-doxyl PC, 12-doxyl PC and 16-doxyl PC) (Figure 4). Fast reorientational motion with rotational correlation times below 1 ns results in three equally spaced sharp lines of similar amplitude. Rotational correlation times in the ns time scale or moderate spatial restriction of the nitroxide reorientational motion are reflected in spectra with increased and different linewidths for the three resonance lines and increased apparent hyperfine splitting. Rotational correlation times above a few tens of ns or strong spatial restrictions result in so-called powder spectra. For all studied samples (Figure 4b, left panel), the EPR spectra show a gradual decrease of the linewidths and apparent hyperfine splitting when comparing 5-, 12- and 16-doxyl PC. The motional restriction is largest for 5-doxyl PC with the nitroxides located close to the lipid head groups and smallest for 16-doxyl PC with the nitroxide located close to the center of the bilayer. The superimposed spectra for the three different sizes of DIBMALPs prove only very small differences revealing that the lipid dynamics are not much influenced by the DIBMALP size (Figure 4b, left panel). The same is true when compared the lipid dynamics in DIBMALPs in presence and absence of *Np*SRII (Figure 4b, right panel). The spectra of 5-doxyl PC do not significantly differ beyond noise, whereas the spectra of 12-doxyl PC and 16-doxyl PC in protein filled DIBMALPs seem to show a slight increase of lipid dynamics compared to empty DIBMALs, as indicated by the slightly different shapes (amplitudes, widths) of the low field peaks (between 343.0 and 343.5 mT).

**Figure 5:**
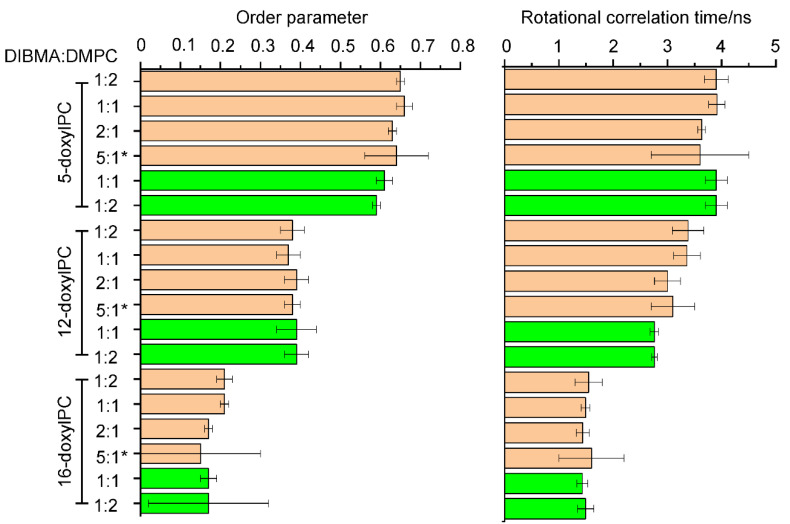
Order parameter S and rotational correlation time τ calculated from the fits of the spectra shown in Figure 4 for empty (brown) and *Np*SRII containing (green) DIBMALPs. * For comparison, the values for empty DIBMALPs prepared with a concentration ratio of 5:1 are given (taken from [45]).

For a quantitative description, we performed spectra simulations based on a model of axial symmetric Brownian reorientation diffusion [47,48], according to Colbasevici et al. [45]. Fit parameters are the rotational correlation times, the inclination of the nitroxide axes with respect to the main lipid diffusion axes represented by the diffusion tilt angle, βD, and the segmental order parameter, S. The order parameter describes the motional restriction of the bound nitroxides, which results from the restoring potential acting on the lipids in the membrane. The values of these parameters are shown in Figure 5. A rigid crystal structure of the membrane is represented by the maximum value of the order parameter, S = 1, whereas membranes in a state of total dynamic disorder are characterized by the lowest possible value, S = 0. For 5-doxyl PC S ranges between 0.59 and 0.66, with the lower values found for DIBMALPs filled with *Np*SRII. The corresponding nitroxide position is located in the most ordered region of the bilayer, in agreement with earlier finding for the lipid dynamics in nanodiscs and liposomes [45]. The presence of *Np*SRII seems to slightly increase the local lipid disorder, whereas the rotational correlation time is not significantly affected by the size of the nanoparticle or the presence of the protein. The values of S for 12-doxyl PC represent intermediate values. The rotational correlation times of the lipids in protein filled DIBMALPs are slightly smaller compared to those of empty DIBMALPs. 16-doxyl PC represents a location of the nitroxide close to the end of the lipid tail and thus in the middle of the bilayer. Its dynamics are less restricted compared to the 5th and 12th positions, as reflected in a rotational correlation time at or below 1.6 ns and order parameters below 0.21. The rotational correlation times do not differ for the present samples within their error margins. The behavior of the order parameter reveals a slightly decreased lipid ordering in the smallest DIBMALPs and in the presence of *Np*SRII.

### 2.3. Coarse-Grained Molecular Dynamics Simulations of Dibmalps Complement the Experimental Results

**Figure 6:**
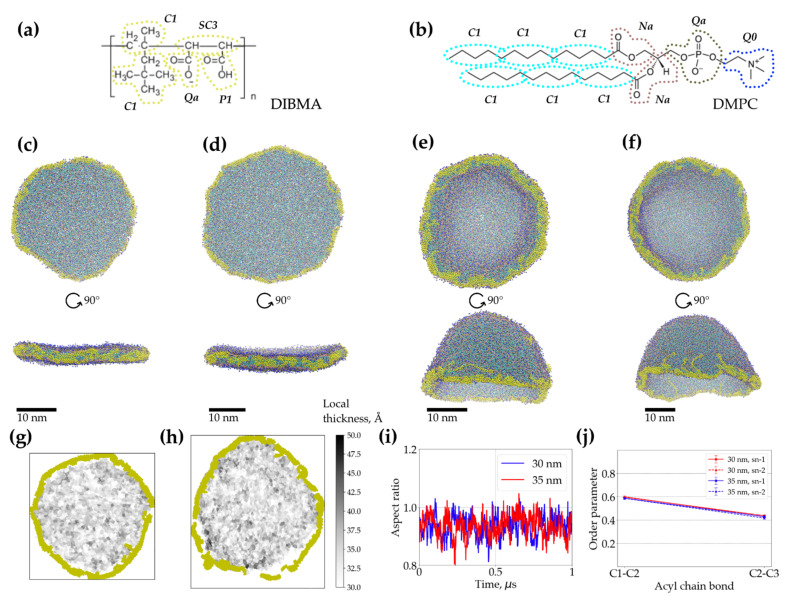
Coarse-grained (CG) molecular dynamics (MD) simulations of DIBMALPs of different diameter. The structural formulae of DIBMA copolymers (**a**) and DMPC lipids (**b**) used in this work. Periodically repeating chemical components of DIBMA polymers are enclosed in brackets and are marked with n. The mapping of all-atom structures to CG MARTINI models is shown with the dotted contours captioned with the corresponding MARTINI CG particle types. The colors of CG particles correspond to those in panels (**c**–**f**). Final structures of 30 nm (**c**), 35 nm (**d**), 40 nm (**e**) and 50 nm (**f**) DIBMALPs after 1-microsecond-long unconstrained MD simulations. Local thickness of 30 nm (**g**) and 35 nm (**h**) DIBMALPs. Ratio of disc dimensions along the first two principal axes of the 30 nm and 35 nm DIBMALP as a function of the simulation time (**i**). Order parameters of the DMPC acyl chains in 30 nm and 35 nm DIBMALPs (**j**).

In order to assess the overall viability of large DIBMALPs and the dynamics of lipids encased in them, we assembled coarse-grained molecular models of DIBMALPs ranging from approx. 30 to 50 nm in diameter, employing the same approach and DIBMA topology (Figure 6a,b) developed before for modeling SMALPs and smaller DIBMALPs (with the diameter of about 10 nm) [45]. The obtained models were subjects for 1 microsecond long unconstrained MD simulations.

The simulations revealed that 30 nm and 35 nm DIBMALPs remain as planar discs throughout the whole simulation time (Figure 6c,d). The aspect ratio of their dimensions along the principal axes indicates that they also remain almost circular in shape (Figure 6i). The analysis of the acyl chain order parameters performed for DMPC in these DIBMALPs of different sizes (Figure 6j) do not show any difference in ordering of the lipid tails in full agreement with the EPR experiments. Also, the local bilayer thickness of both 30 and 35 nm DIBMALPs (3.3 ± 0.7 nm and 3.2 ± 0.8 nm, respectively) match the experimental values obtained by means of AFM (see Figure 3 and Figure 6g,h).

However, the behavior of the larger preformed DIBMALPs with the diameter of 40 and 50 nm during the unconstrained MD simulations was strikingly different to what we observed for 30 and 35 nm DIBMALPs. While the preformed discs are initially planar, they experience large undulations during the simulations and eventually bend into a cup-shaped structure (Figure 6e,f). The curved conformation of 40 and 50 nm DIBMALPs precluded the analysis of the order parameters in this case.

### 2.4. The Photocycle of NpSRII in DIBMALPs Depends on the Protein-to-Lipid Ratio

**Figure 7:**
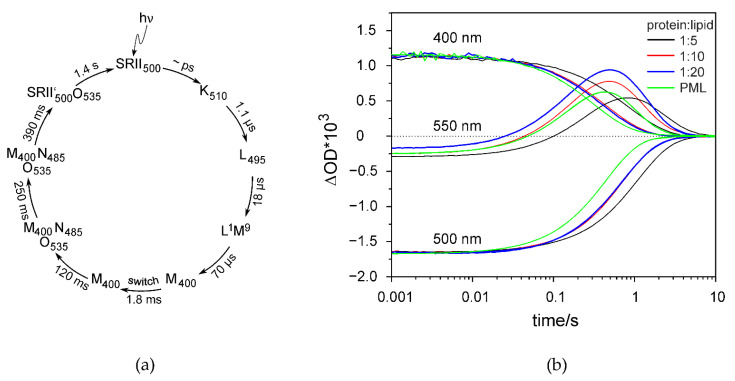
The photocycle of *Np*SRII in DIBMALPs depends on the protein-to-lipid ratio. (**a**) *Np*SRII photocycle characterized by transient changes in the optical absorption spectrum of the retinal chromophore. Subscripts indicate the wavelength of maximum absorption of the intermediates in nm (adapted from [51]). Time constants for the transitions between these intermediates are given according to the values for wildtype *Np*SRII reconstituted in purple membrane lipids [55]. (**b**) Transient optical absorption changes of *Np*SRII in DIBMALPs (DIBMA/lipid ratio 1:1) were recorded at 400 nm (M-intermediate), 500 nm (initial state) and 550 nm (O-state). The *Np*SRII-to-lipid ratio was varied, 1:5 (black), 1:10 (red) and 1:20 (blue). The fitted exponential curves are exemplarily shown for 1:20 as thin lines, which are indistinguishable from the measured transients. Maximum values of transients for 400 and 500 nm were normalized to the corresponding values of sample 1:20. For comparison, the traces of *Np*SRII in native purple membrane lipids (PML) are given (green) (data taken from [38]).

Sensory rhodopsin, *Np*SRII, an archaebacterial photoreceptor from *Natronomonas pharaonis,* belongs to the family of seven transmembrane helix proteins and is thus related to G-protein coupled receptors [49]. It mediates the photophobic response of *N. pharaonis* to green-blue light under aerobic conditions [50]. Activation by light leads to isomerization of the retinal chromophore, which is followed by a sequence of spectroscopically characterized intermediates, named K, L, M, N and O, leading back to the initial ground state (Figure 7a) [51]. The transition between the two M states (“switch”) is accompanied by an outward movement of helix F [52,53], which was shown to trigger a conformational change of the associated transducer molecule. The photocycle kinetics and the corresponding conformational transitions of *Np*SRII are sensitive to changes of environmental conditions like lipid composition, temperature and pH [5,51,54]. This is especially true for the transitions following the M-decay. Hence, these transitions are examined here to prove protein function and determine possible effects of protein–polymer or protein–protein interaction. Light-induced transient absorption changes were recorded for three characteristic wavelengths (Figure 7b). The decay of intermediate M was monitored at 400 nm, the formation and decay of the O-state was followed at 550 nm, and the recovery of the initial state was recorded at 500 nm. The time constants from the multi-exponential fits (0.25 ± 0.01 s, 0.57 ± 0.03 s, 1.2 ± 0.1 s for protein/lipid ratio of 1:10) resemble those of *Np*SRII reconstituted in purple membrane lipids [38,55] and show that *Np*SRII retains its functionality in DIBMALPs. Strikingly, the increase of the weight lipid-to-protein ratio from 1:5 to 1:10 or 1:20 leads to a significant acceleration of the photocycle, most probably due to a decreased protein–protein interaction in DIBMALPs with increased lipid content (see Discussion).

## 3. Discussion

In this work, we combine different biophysical approaches to compare the size distribution, particle homogeneity and lipid dynamics of DIBMALPs prepared at different DIBMA/DMPC weight ratios and in presence and absence of *Np*SRII. The DLS results show that an increase of the DIBMA content during the preparation leads to a corresponding decrease of the overall size of DIBMALPs for the tested DIBMA/DMPC compositions. These data are consistent with published values [24]; in this work, the authors observed DIBMA/DMPC particles with a hydrodynamic diameter of approximately 35 nm at a DIBMA/DMPC molar ratio of 0.08 and of approximately 18 nm at a DIBMA/DMPC molar ratio of 0.20. Thus, our DLS data, in agreement with these previous results [24], show that varying weight ratios of DIBMA/DMPC controls the overall particle size in a range of about 30–50 nm. EM imaging represents individual DIBMALPs with sizes corresponding to the DLS values. Interestingly, TEM micrographs show the presence of stacked discs. These discs seem to be similar to those observed in negative-stained samples of phospholipid bilayers from purified plasma apolipoproteins and synthetic phospholipids. These structures have a regular repeat distance close to the thickness of a phospholipid bilayer [56,57]. Similar stacks have been observed also in nanodiscs [2]. Since negative staining can cause artefacts owing to fast dehydration of the sample [58], we combined negative staining with plunge-frozen samples in cryo-EM. Cryo-EM enables to avoid such artefacts because the rapid cryo-immobilization (plunge freezing used in this work) preserves the aqueous environment of the specimen whilst also preventing its possible damage [59]. We used AFM because of its high axial resolution that proved the native-like thickness of the assembled DIBMALPs (Figure 3), in agreement with previous data obtained for SMALPs [46].

The MD simulations confirm the viability and overall stability of planar DIBMALPs with the dimeter of 30 and 35 nm, whereas larger DIBMALPs tend to curve and form cup-shaped structures during simulations (Figure 6e,f). Similar structures were previously shown to represent intermediates of simulated bilayer-to-vesicle [60,61] and bicelle-to-vesicle transitions [62], which encourages us to speculate that our simulations (restricted by 1 microsecond each due to the limited computational resources) would also end up in a vesicle-shaped conformation if continued. At the same time, the wrapping of DIBMA-stabilized planar bilayers to cup-like structures was observed starting from a much larger number of lipids (more than 3000, see Table 2 in Methods) as compared to the plain lipid bilayers/bicelles where the bilayer-to-vesicle transition took place in systems consisting of as few as 512 lipids [60]. This process is supposed to be enthalpically unfavorable and entropically-driven; the conversion to vesicles decreases the solvent-accessible hydrophobic area of the bilayer [60]. Interactions between DIBMA and lipids may provide an additional enthalpic contribution and screen the hydrophobic edges of the bilayer. Both effects would stabilize the planar DIBMALPs. We are planning to investigate these effects systematically in future work.

Apart from the limitations of the utilized CG model [63], the apparent discrepancy in nanoparticle formation between the largest analyzed DIBMALPs (with DIBMA/lipid ratio 1:2) observed in the experiments and in our in silico simulations may be due to the different actual proportion of polymers and lipids in DIBMALPs, which is hard to control and follow. Another difference between the experimental and model systems was in the mechanism of DIBMALPs formation. While in the model system DIBMA molecules were added to a planar lipid bilayer, in the experiments, DIBMA molecules were added to spherical liposomes. Earlier we showed that copolymers extract whole lipid patches to form lipid nanoparticles [44]. If so, the DIBMA molecules would compete for lipids at higher DIBMA-to-lipid ratios. In addition, the average area of available free lipid patches would be smaller in case of proteoliposomes. Therefore, one could expect that the size of DIBMALPs would decrease with increased DIBMA-to-lipid and protein-to-lipid ratios, in agreement with experimental observations; see Figure 1b.

EPR spectroscopy shows that lipid dynamics does not depend on the size of the DIBMALPs in the studied size range in agreement with the results of the MD simulations. Thus, polymer–lipid interactions that might affect lipid dynamics at the polymer–lipid interface [45] play a negligible role. Similarly, the presence of *Np*SRII in DIBMALPs does not considerably change the average lipid dynamics. In contrast, different protein-to-lipid ratios in the preparation of *Np*SRII containing DIMALPs affect the photocycle kinetics. With 1600 lipids contained in a DIBMALP of 25 nm diameter (calculated from the values given in Table 2) and with the assumption that the treatment of the proteoliposomes with DIBMA does not change the protein-to-lipid ratio, the average number of *Np*SRII molecules encased in a single DIBMALP prepared with a protein-to-lipid ratio of 1:5 (*w*/*w*) (corresponding to 1:172 (mol/mol)) is estimated to be nine or fewer. For a protein-to-lipid ratio of 1:20 the average occupation number is two, thereby reducing significantly possible protein–protein interaction or formation of oligomers. The photocycle of *Np*SRII is known to be modulated by protein–lipid interaction [5,38] and protein–protein interaction [64]. In particular, the interaction of *Np*SRII with its transducer *Np*HtrII accelerates the decay of M and O intermediates due to a more constraining *Np*SRII molecule, in which the conformations of M and N are destabilized [64]. *Np*SRII has been reported to form trimers [65]. It is thus tempting to speculate that the observed dependence of the photocycle on the lipid-to-protein ratio in the prepared DIBMALPs is due to a different degree of *Np*SRII trimerization or higher oligomerization. The present results thus indicate a possible expansion of the applicability of the DIBMALP technology for studies of membrane protein–protein interaction and oligomerization in a constrained environment.

## 4. Materials and Methods

### 4.1. Chemicals

1,2-Dimyristoyl-*sn*-glycero-3-phosphocholine (DMPC) was a kind gift from Lipoid (Ludwigshafen, Germany). 1-Palmitoyl-2-stearoyl-(5-doxyl)-sn-glycero-3-phosphocholine (16:0-5 doxyl PC), 1-palmitoyl-2-stearoyl-(12-doxyl)-sn-glycero-3-phosphocholine (16:0-12 doxyl PC), 1-palmitoyl-2-stearoyl-(16-doxyl)-sn-glycero-3-phosphocholine (16:0-16 doxyl PC) were bought from Sigma-Aldrich (St. Louis, MO, USA). DIBMA copolymer, commercially available under the trade name PureCube DIBMA 10 in TRIS (lyophilized in Tris-buffer, pH 7.5), was purchased from Cube Biotech (Monheim am Rhein, Germany). N-Dodecyl-ß-D-maltoside (DDM) was acquired from Anatrace (Affymetrix, Cleveland, OH, USA). Isopropyl-β-D-thiogalactopyranosid (IPTG) was bought from Carl Roth (Karlsruhe, Germany). EDTA-free complete protease inhibitor cocktail was procured from Roche Life Science (Mannheim, Germany). Ni-NTA superflow agarose was purchased from Qiagen (Hilden, Germany). All other reagents were of analytical grade.

Protein concentrations were determined using an UV-VIS spectrophotometer (UV-2450, Shimadzu Corporation, Kyoto, Japan). The concentration of *Np*SRII was determined by using the known extinction coefficient of 50,000 M^−1^ cm^−1^ at λ = 280 nm

### 4.2. Protein Expression and Purification

For purification purposes, *Np*SRII has a C-terminal 6xHis-tag. *Np*SRII-His was expressed in *Escherichia coli* BL21 (DE3) cells and purified according to [66,67] with minor modifications. Briefly, transformed cells were grown in Luria–Bertani (LB) medium containing 50 mg/mL kanamycin at 37 °C to an optical density OD_580_ of 1.0. The overexpression of the protein was induced by addition of IPTG to a final concentration of 0.5 mM. 10 μM all-trans retinal (Sigma, St. Louis, MO, USA) was also added. After an induction period of 3 h at 37 °C, cells were harvested (4200× *g*; 15 min; 4 °C), washed and resuspended (1/100 culture volume) in 150 mM NaCl, 25 mM NaPi (pH 8.0), 2 mM EDTA buffer containing a protease inhibitor mix. Cells were disrupted by sonication (Branson Sonifier II W-250, Heinemann, Germany). Membranes were isolated by centrifugation (50,000× *g*; 1 h; 4 °C), and solubilized in buffer A (300 mM NaCl, 50 mM NaPi (pH 8.0), 2% (*w*/*v*) DDM) overnight at 4 °C. Solubilized membrane proteins were isolated by centrifugation (50,000× *g*; 1 h; 4 °C) followed by chromatography using Ni-NTA superflow material which was pre-equilibrated with buffer B (300 mM NaCl, 50 mM NaPi (Na_2_HPO_4_/NaH_2_PO_4_) pH 8.0, 0.05% (*w*/*v*) DDM). Non-specifically bound proteins were removed by washing extensively with buffer B containing 30 mM imidazole. His-tagged protein was eluted with buffer B containing 200 mM imidazole. Fractions containing the desired protein were pooled and dialyzed against buffer C (500 mM NaCl, 10 mM Tris (pH 8.0), 0.05% (*w*/*v*) DDM)) to remove imidazole. If not used directly for reconstitution, protein samples were flash frozen and stored at −80 °C.

### 4.3. Liposome Preparations

Powdered DMPC was dissolved in chloroform to a final lipid concentration of 7.37 mM and if desired mixed with 1 mol% of lipid spin label. Chloroform was evaporated under a stream of nitrogen gas. The resulting lipid film was dried under vacuum for at least 2 h. The dried lipids were suspended in 50 mM Tris (pH 7.5), 200 mM NaCl (buffer D) and vortexed. Subsequently, the multilamellar liposome suspension underwent five freeze–thaw cycles (N_2_/water bath at 37 °C), and, if not used directly, was stored in aliquots at −80 °C. Before reconstitution, the suspension of liposomes was extruded 31 times through polycarbonate membranes of 100 nm pore size using a Mini-Extruder Set (Avanti Polar Lipids, Alabaster, AL, USA) to get unilamellar vesicles.

### 4.4. Proteoliposome Preparation

Proteoliposomes were prepared as published before [29] with minor modifications. Briefly, preformed liposomes were equilibrated by stirring for 3 h with n-dodecyl-β-D-maltopyranoside (DDM) at room temperature with a lipid to detergent mixing ratio of 1:1 (mol/mol). Before adding the protein solution, the lipid–detergent mixture was placed in an ultrasonic bath at room temperature for 10 min. Then *Np*SRII was mixed with the equilibrated with DDM liposomes at a protein-to-lipid weight ratio of either 1:5, 1:10 or 1:20 (*w*/*w*) and incubated for 30 min at room temperature with gentle shaking. To remove the detergent, the reconstitution of the *Np*SRII in liposomes was carried out using hydrophobic polystyrene beads, SM-2 Bio-Beads (Bio-Rad Laboratories, Munich, Germany). These hydrophobic polystyrene beads were washed thoroughly beforehand with methanol and water followed by buffer D. The Bio-Beads were added to the protein–lipid detergent solution at a ratio of 10:1 (*w*/*w*) of the wet Bio-Beads to detergent. The samples were incubated for one hour at room temperature. A new portion of Bio-Beads was then added, and the samples were incubated at 4 °C overnight with gentle shaking. After removing the Bio-Beads, the proteoliposomes were collected by centrifugation (15,800× *g*; 30 min; 4 °C). Finally, they were resuspended in buffer A to a final lipid concentration of 7.4 mM.

### 4.5. Preparation of DIBMALPs

DIBMALPs were prepared as published before [13]. Briefly, to form DIBMA/lipid particles, a 4% (*w*/*v*) aqueous solution of DIBMA copolymer was added dropwise to the liposome suspension to get a copolymer/lipid weight ratio of 1:2, 1:1 or 2:1 at a final lipid concentration of 0.2% (3 mM) (a copolymer/lipid molar ratio of 0.033; 0.067; 0.133, corresponding to a final polymer concentration of 0.1%, 0.2% and 0.4% (*w*/*v*), respectively). The assembly mixture was allowed to equilibrate for 1 h at room temperature and then for 16 h at 4 °C. The resulting samples were centrifuged (126,000× *g*; 30 min; 4 °C) to remove aggregates. If necessary, the samples were concentrated using 3 kDa MWCO Vivaspin 500 ultrafiltration devices (Sartorius, Epsom, UK).

### 4.6. Preparation of DIBMALPs Containing NpSRII

To form DIBMALPs containing *Np*SRII (in the following abbreviated as *Np*SRII-DIBMALPs), a 4% (*w*/*v*) aqueous solution of the DIBMA copolymer was added dropwise to the suspension of proteoliposomes to obtain a final copolymer/lipid weight ratio of 1:1 or 2:1. The mixture was then equilibrated for 1 h at room temperature and then at 4 °C for 16 h. The samples were centrifuged (126,000× *g*; 30 min; 4 °C) to remove the unsolubilized proteins. The resulting samples were then incubated for one hour at room temperature with gentle agitation with Ni^2+^-NTA agarose (Qiagen), which had previously been equilibrated with buffer E (300 mM NaCl, 50 mM NaPi (Na_2_HPO_4_/NaH_2_PO_4_), pH 8.0). Atypically bound material was removed by washing with 40 mM imidazole in buffer E. DIBMALPs containing His-tagged *Np*SRII were eluted with 200 mM imidazole in buffer E and if necessary dialyzed against buffer D. At both 1:1 and 2:1 copolymer/lipid weight ratios, the yield of affinity purified *Np*SRII after extracting from proteoliposomes was slightly more than 20%.

### 4.7. Dynamic Light Scattering

DLS measurements were performed on a Zetasizer Nano ZS (Malvern Instruments, Worcestershire, UK) at 550 nm and 25 °C. Data represent the average of three sets of 14 runs of 10 s each. The particle size distribution was obtained by using the ZETASIZER software package Ver. 7.02. under the assumption that DIBMA/lipid nanoparticles were spherically shaped.

### 4.8. Transmission Electron Microscopy

Freshly prepared DIBMALPs (2 mg lipid/mL) were diluted 1:100 in buffer D, and 4 µL were applied onto negatively glow-discharged carbon-coated 400-mesh copper grids (300-mesh formvar/carbon-coated) (Ted Pella, Redding, CA, USA) for 1 min. Excess liquid was removed by blotting with filter paper. The grid was washed twice shortly with buffer and stained 4 min with 2% uranyl acetate and blotted. Digital micrographs were collected using a JEM2100Plus Transmission Electron Microscope (JEOL, Akishima City, Japan) operated at 200 kV equipped with a XAROSA CMOS 20 Megapixel Camera (EMSIS GmbH, Muenster, Germany).

### 4.9. Cryo-Transmission Electron Microscopy

DIBMALPs (2 mg lipid/mL) were diluted 1:10 in buffer D and 4 µL of DMPC/DIBMA suspension was placed on glow-discharged holey copper grid (Quantifoil R2/2 200 Mesh). Grids were blotted for 3 s at 10 °C, 80% humidity, and frozen in liquid ethane using EM GP2 plunge freezer (Leica, Wetzlar, Germany). Grids were transferred into a JEOL JEM2100Plus Transmission Electron Microscope operating at 200 kV. Digital micrographs were recorded manually with a XAROSA CMOS 20 Megapixel Camera.

### 4.10. Atomic Force Microscopy

For AFM imaging, 250 µL of a solution of DIBMALPs was put in contact with freshly cleaved mica, previously glued to a coverslip, and diluted with additional 250 µL of buffer (50 mM Tris, 200 mM NaCl, pH 7.5). The DIBMALP sample was incubated for 10 min, then mica was washed 3 times with 500 µL of the buffer and then allowed to equilibrate at room temperature before analysis. DIBMALPs were imaged using a JPK NanoWizard II system (JPK Instruments, Berlin, Germany) mounted on an Olympus IX71 Inverted Microscope (Olympus Corporation, Tokyo, Japan). Intermittent contact (IC or tapping) mode images were taken using an SNL-10 V-shaped silicon nitride cantilevers (Bruker AFM Probes, Camarillo, CA, USA) with a typical spring constant of 0.06 N/m. The cantilever oscillation was tuned to a frequency between 3 and 10 kHz, and the amplitude was set between 0.3 and 0.7 V. The amplitude was varied during the experiment to minimize the force of the tip on the particles. The scan rate was set to 0.3–0.7 Hz. Images were processed by the JPK processing software, applying a smoothing function. Lipid nanoparticle thickness was measured based on the height profiles from the mica (taken as 0 nm).

### 4.11. Electron Paramagnetic Resonance Spectroscopy

EPR measurements were performed as described previously [68]. Briefly, room temperature continuous wave (cw) EPR spectra were recorded on a home-built EPR spectrometer equipped with a dielectric resonator (Bruker Biospin, Ettlingen, Germany). Glass capillaries of 0.9 mm inner diameter were filled with sample volumes of ~20 μL. The measurements were recorded at (9.686 ± 0.005) GHz in a B-field range from 340 mT to 352 mT. The field modulation was set to 0.22 mT. The temperature during the measurements was 295 K.

### 4.12. EPR Spectra Simulations

Other variables, which were allowed to vary during the fittings, were the rotational diffusion tensor of axial symmetry defined by R_⟂_ and R_∥_, the coefficient of the orienting (restoring) potential, C_20_, from which MultiComponent calculates the order parameter S, and an isotropic linewidth, W. The errors were determined by repeating the fits with different starting parameters of the angle β_D_. For this purpose, the value of β_D_ was changed by ±5° around the best-fit value.

### 4.13. Transient Optical Absorption Spectroscopy

Transient optical absorption experiments were carried out as described previously [55]. A 50 W halogen lamp with an infrared cutoff filter (KG-2) and 400 nm, 500 nm or 550 nm interference filters illuminated the sample-filled quartz cuvette inside a sample holder, which was temperature-controlled to 298 K. The transmitted light was passed through a second interference filter and detected by a photodiode. A flashlight with a flash duration of ~80 µs equipped with a 475 nm edge filter provided excitation perpendicular to the transmission beam. The amplified signal was recorded with an analog-to-digital converter connected to a standard PC. For the transitions between the late photocycle intermediates (t > 2 ms) studied here, the kinetics determined by flashlight excitation were indistinguishable from those determined by pulsed laser excitation [68]. The transient absorption changes were recorded on samples buffered in 50 mM Tris, pH 7.50, 200 mM NaCl at concentrations of approximately 5 μM.

### 4.14. Molecular Dynamics Simulations

In order to simulate large DIBMALPs, we used the coarse-grained model of the DIBMA polymer (consisting of 52 units with the 1:1 diisobutylene/maleic acid ratio) developed by us recently [45] within the framework of the popular MARTINI force field [63]. The standard MARTINI library contains the CG force field parameters for DMPC.

We used Gromacs 2018.1 for all MD simulations [69]. The DIBMALPs of various sizes (see Table 2 for the overview of all simulated systems) were assembled starting from a circular DMPC lipid patch created by the insane.py script [70] surrounded by DIBMA polymers in the amount sufficient to form a single layer of polymers at the rim of the lipid patch (estimated from the contour length of a single polymer and the lipid patch perimeter).

Energy minimization using the steepest descent algorithm prefaced each equilibration simulation run in the NVT ensemble (simulation time equaled 1 μs) maintained by the V-rescale thermostat (T = 320 K, τ_t_ = 1.0 ps) during which the phosphate groups of lipids were constrained in the plane perpendicular to the normal of the lipid patch using a harmonic potential with the force constant = 1000 kJ/mol/nm^2^.

The production simulations were run for 1 μs each in the NPT ensemble using the same thermostat and the Parrinello–Rahman barostat (time constant = 12.0 ps, compressibility = 3 × 10^−4^ bar^−1^, as recommended in [71], applied isotropically). All CG simulations were performed in the explicit solvent with the standard water model, at 0.15 M concentration of NaCl. The reaction field approach was used to treat the long-range electrostatics with the relative permittivity of 15 and the Coulomb cut-off of 1.1 nm [71]. A time step of 20 fs was utilized for all simulations. The Verlet pair-lists cutoff scheme was used and the neighbor list was updated every 20 steps. Periodic boundary conditions were applied in all simulations.

For analysis, the DIBMALPs were aligned such that their center-of-weight motion was removed, and their principal axes were aligned with the coordinate axes at every trajectory step. In-house Python scripts exploiting MDAnalysis [72] were used for the alignment, calculation of the aspect ratio along the first principal axes and analysis of the order parameters of lipid acyl chains. Local thickness was calculated as in [73].

## Figures and Tables

**Table 1:** z-Average diameter, z, of DIBMALPs determined by DLS at different DMPC/DIBMA ratios for preparations in absence and presence of *Np*SRII (average values of at least two repetitions). * In addition, the values from [45] are given for comparison, and were determined at a concentration (DIBMA:lipid) of 5:1.

DIBMA/DMPC *w*/*w*	Z-Average Hydrodynamic Diameter [nm]	
	*−Np*SRII	+*Np*SRII
1:2	53.9 ± 0.4	36.3 ± 4.6
1:1	41.5 ± 2.0	25.9 ± 1.0
2:1	26.5 ± 1.6	-
5:1 *	26.2 ± 3.0	-

**Table 2:** Overview of the simulated systems.

DIBMALP Diameter, nm	Number of DMPC	Number of DIBMA	Box Size, nm × nm × nm
30	2322	26	39.5 × 39.5 × 39.5
35	3162	30	45.7 × 45.7 × 45.7
40	4138	35	51.7 × 51.7 × 51.7
50	6520	44	60.8 × 60.8 × 60.8

## Data Availability

All data generated or analyzed during this study are included in this published article.
